# Application of system dynamics and participatory spatial group model building in animal health: A case study of East Coast Fever interventions in Lundazi and Monze districts of Zambia

**DOI:** 10.1371/journal.pone.0189878

**Published:** 2017-12-15

**Authors:** Chisoni Mumba, Eystein Skjerve, Magda Rich, Karl M. Rich

**Affiliations:** 1 The University of Zambia, School of Veterinary Medicine, Department of Disease Control, Lusaka, Zambia; 2 Norwegian University of Life Sciences, Department of Food Safety and Infection Biology, Section for Epidemiology and Statistics (Epicentre), Oslo, Norway; 3 University of Brighton, College of Arts and Humanities, Grand Parade, United Kingdom; 4 International Livestock Research Institute (ILRI), East and Southeast Asia Regional Office, Ba Dinh District, Hanoi, Viet Nam; University of Vermont, UNITED STATES

## Abstract

East Coast Fever (ECF) is the most economically important production disease among traditional beef cattle farmers in Zambia. Despite the disease control efforts by the government, donors, and farmers, ECF cases are increasing. Why does ECF oscillate over time? Can alternative approaches such as systems thinking contribute solutions to the complex ECF problem, avoid unintended consequences, and achieve sustainable results? To answer these research questions and inform the design and implementation of ECF interventions, we qualitatively investigated the influence of dynamic socio-economic, cultural, and ecological factors. We used system dynamics modelling to specify these dynamics qualitatively, and an innovative participatory framework called spatial group model building (SGMB). SGMB uses participatory geographical information system (GIS) concepts and techniques to capture the role of spatial phenomenon in the context of complex systems, allowing stakeholders to identify spatial phenomenon directly on physical maps and integrate such information in model development. Our SGMB process convened focus groups of beef value chain stakeholders in two distinct production systems. The focus groups helped to jointly construct a series of interrelated system dynamics models that described ECF in a broader systems context. Thus, a complementary objective of this study was to demonstrate the applicability of system dynamics modelling and SGMB in animal health. The SGMB process revealed policy leverage points in the beef cattle value chain that could be targeted to improve ECF control. For example, policies that develop sustainable and stable cattle markets and improve household income availability may have positive feedback effects on investment in animal health. The results obtained from a SGMB process also demonstrated that a “one-size-fits-all” approach may not be equally effective in policing ECF in different agro-ecological zones due to the complex interactions of socio-ecological context with important, and often ignored, spatial patterns.

## Introduction

East Coast fever (ECF) is an important disease of cattle caused by a protozoan parasite *Theileria parva* which is transstadially transmitted by a three-host tick *Rhipicephalus appendiculatus* [1]. The disease is of uttermost economic importance in Eastern, Central, and Southern Africa where it causes severe direct and indirect economic losses among cattle farmers [2,3]. In Zambia, it is economically the most significant production disease. However, it is classified as a management disease, which places the responsibility on farmers to control it [4,5]. In 1994, an estimated 10,000 cattle died from ECF in Zambia [6].

ECF control measures include immunisation (strictly provided by Government at a fee), chemotherapy, and tick control through the use of acaricides. Tick control is the responsibility of farmers, although the government provides dip tanks [7]. Despite disease control efforts by several donor-funded projects (e.g., ASVEZA program by the Belgian government) [1], farmers, and the Government of Zambia, ECF cases are on the increase in the Eastern province (where the disease is endemic) and other provinces of Zambia [8]. We further confirmed the increase of ECF cases by a survey which we conducted (n = 699) to identify key diseases affecting traditional cattle farmers [9].

However, there is a lack of research to identify the causes behind the increase in ECF cases. Many studies in Zambia and globally have discussed ECF-related issues such as the quantification/modelling of its economic impacts [10–14], epidemiology and genetic diversity [7,15,16]. Other studies include: risk analysis of ECF transmission [4], trend analysis using GIS [2] and linear stochastic modelling of its epidemiology [17]. However, there is limited research that teases out the drivers of disease response and control. Potential drivers include socio-economic, cultural, or socio-ecological factors such as the social class of traditional cattle farmers in the community, attitudes of traditional cattle farmers, community/societal norms, and the environment [18]. It is important to understand these factors and the extent that they interact in ways that could influence the implementation and effectiveness of ECF policy. We hypothesise that socio-ecological dynamics significantly influence the ability of current policy measures to control ECF in Zambia. Consequently, policies to control ECF need to involve not only technical solutions but also consider their socio-economic and ecological context.

To test this hypothesis, we used a qualitative system dynamics modelling approach. System dynamics, or SD, applies the modern theory of nonlinear dynamics and personal construct theory to solve problems based on the dynamic behaviour observed in complex systems [19,20]. An important aspect of SD models is the concept of feedback in which shocks (as well as planned changes in practices, policies, etc) to a system can have dynamic, unanticipated consequences on system behaviour. We describe feedback as interactions among components of the system and how they reinforce or self-correct system behaviour [19]. Through model building, these feedback structures can be identified and quantified, which allows practitioners to observe the influence of specific drivers in a system.

In an animal health context, and particularly in the context of ECF in Zambia, it is important to recognise that the nature of disease and disease processes are just one part of the broader livestock system that includes market, socio-economic, and environmental factors. Household decisions made on the basis of social obligations, conventions, ethnic rivalry, or other household needs further thwart the technical, top-down efforts of policymakers to control disease. These structures, and their interactions produce different patterns of disease endemicity over time, and which can be influenced by the introduction of public policies aimed at mitigating disease incidence [21]. From a policy perspective, SD models provide decision makers with insights on how interrelated factors /drivers influence disease patterns. These insights are typically missing among decision-makers, and we argue that a greater appreciation could help them develop policies that more effectively integrate technical solutions with socio-ecological interventions (e.g. farmer awareness, education, or specific socio-economic policies).

SD models can be developed using participatory processes, which improves their accessibility and internal validity [20]. In this study, we used a recent innovation in participatory processes called spatial group model building (SGMB) [22]. SGMB uses geographical information system (GIS) concepts and techniques that allow capturing stakeholders to identify the spatial phenomenon directly on physical maps and describe how the phenomenon interact in the form of qualitative SD models. SGMB improves the quality of information received from stakeholders and grounds it spatially by providing more targeted details on the “where” (i.e., the location) of problems in question. For diseases like ECF, the setting of the system, including the location of farms, socio-economic characteristics of farmers, the location of veterinary information, movement patterns of animals, land use, climatic factors, etc., and their interactions, all affect the dynamics of disease evolution. They further reflect important leverage points for policy that could influence the effectiveness of interventions. SGMB provides a platform to tease out this information to develop more appropriate, locally relevant models. In this study, we used SGMB to highlight and contrast differences in disease drivers in two cattle producing regions in Zambia.

The objectives of this study were twofold. First, we qualitatively investigate the potential influence played by dynamic socio-economic and ecological factors in order to improve the design and implementation of ECF interventions. Second, we demonstrate the applicability of system dynamics and SGMB in addressing complex animal health problems.

## Materials and methods

We obtained ethical clearance (consistent with Norwegian University of Life Sciences policy) from Excellence in Research Ethics and Science (ERES) Converge, reference number “2016-Nov-006”.

We followed standard practices of the systems dynamics modelling process which includes dynamic problem articulation; dynamic hypothesis formulation; model conception, construction, and simulation; and policy design and evaluation [19,23]. In this paper, we went through the first four qualitative steps, and plan implementation of the last three quantitative steps for future studies. We hypothesised that the socio-economic, cultural and ecological context plays a critical role in shaping the response to ECF, and consequently will influence the effectiveness of ECF interventions. We identified the complex and chronic problem through a face-to-face interview-based survey (n = 699) whose results we have described in detail in a paper we recently published [9]. We then engaged stakeholders in the beef value chain to get a broader view of the problem. We used spatial group model building to understand the complexity of the problem with regard to the physical space in which it occurs. We finally used system dynamics modelling software (Stella Professional, see http://www.iseesystems.com) to visualise and understand how the various sub-systems (socio-economic, cultural, ecological factors) associated with the problem interact and use this characterization to identify leverage points for policy. The leverage points were used to inform on policy decisions that could better address the complex problem, avoid unintended consequences, and achieve lasting results.

### 2.1 System dynamics modelling

System dynamics is a computer-aided approach to policy analysis and design. The approach is interdisciplinary and applied to dynamic problems that arise from complex social, managerial, economic, or ecological systems [21]. System dynamics models can either be qualitative or quantitative. Quantitative models employ computer simulation on a parameterised model to test hypotheses about the relationships between the system structure and system behaviour [24]. Quantitative SD models are systems of nonlinear differential equations that specify behaviour and relationships in complex systems [25]. However, rather than representing such mathematical complexity through programming code, SD uses graphical features to intuitively represent these mathematical formalisations that are defined in the background, which allows for easier of communication with and use by non-technical audiences. In a qualitative analysis, the focus is on the structure of the system and to identify the feedback effects that could drive system behaviour over time. This paper focuses on the qualitative and diagramming aspects of the approach.

**Fig 1:**
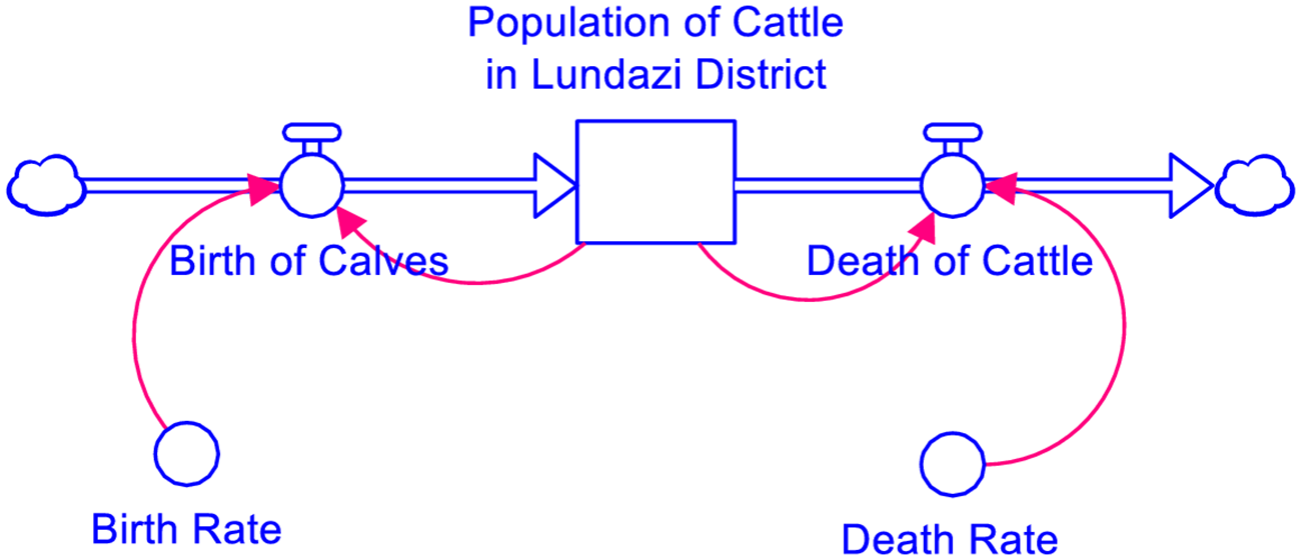
Cattle population stock and flow diagram (developed by authors).

The language of system dynamics includes stocks, flows, and parameters, which we illustrated in a simple model of cattle population dynamics in Fig 1. Stocks (the rectangular shape in Fig 1) represent anything that accumulates or depreciates over time, e.g., people, diseases, or currency. The number of animals on a farm at a specific time period (e.g. January 2016) would be an example of a stock in this context. The quantity found in a stock is changed by the inflow or outflow (the thick arrows in Fig 1) of goods or services from that stock; collectively, these inflows or outflows are referred to as “flows”. For example, the birth of calves increases the population of cattle while deaths reduce the population. A parameter determines the speed at which flows occur. For example, the birth rate (percentage of births in a year) will determine the rate at which the cattle population increases as shown in Fig 1.

In this study, our focus was on the qualitative structure of the system to identify the drivers of ECF control. We constructed the models using Stella Professional software, with the aim to later parameterise for quantitative analysis in future research.

### 2.2 Group model building

System dynamics models can be developed through a variety of means. Often, primary and secondary data are used to develop SD models, but SD models can also be built through participatory processes such as Group Model Building (GMB). GMB engages stakeholders directly in the process of model conceptualisation, formulation, analysis, and decision-making using a series of focus group meetings [24,26]. Researchers and practitioners have refined GMB methods and formalised the process through the use of focus group “scripts” [27]. GMB provides a bottom-up process of policy formulation and prioritisation that takes local needs into account [28]. In application to animal health, GMB has the advantage of bringing together diverse stakeholders from the livestock value chain to address common problems from different vantage points and serves as a platform for the joint development of solutions. GMB has been used to develop models and conduct policy analysis in the livestock sector, but its use has been limited [29–31].

**Fig 2:**
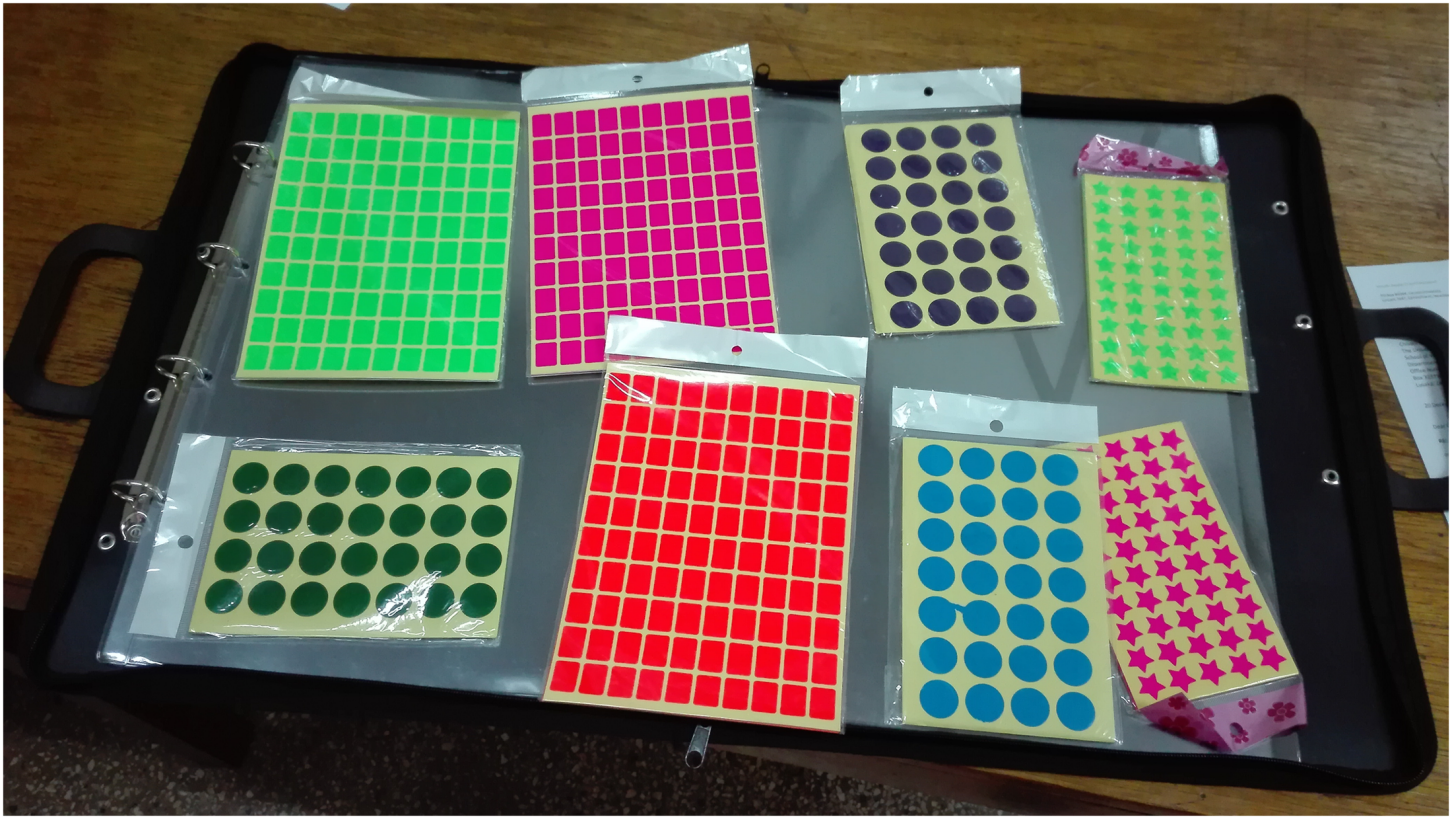
SGMB facilitation tool LayerStack.

In this study, we used a recently developed participatory technique called spatial group model building (SGMB) [22], to collect data from stakeholders in the beef value chain in Lundazi and Monze districts to develop our qualitative system dynamics models. SGMB is a modification of standard group model building (GMB) that aims to elucidate how land patterns and the environment interrelate and form potential feedback loops with other system factors. SGMB uses maps and GIS techniques to facilitate discussion of these types of spatial phenomena. We used a physical facilitation tool called LayerStack (developed at Lincoln University in New Zealand by two of the study authors). LayerStack uses a series of transparent plastic acetates overlaid on a map of the region of interest. Each acetate denotes a data layer similar to a computer-based GIS. Through LayerStack, stakeholders can better visualise spatial factors of a particular research problem, thus easing the facilitation process (Fig 2). LayerStack does not require the use of computers, projectors, flip charts, or whiteboards like GMB processes and thus enables SGMB to be done in any environment (e.g. outside, under a tree, etc.) [22]. Therefore, it is well suited for developing countries such as Zambia where access to more technology-focused facilities do not exist. Like standard GMB, SGMB creates awareness and motivation among the stakeholders taking part in this decision-making process, and the visualisation improves the quality and efficiency of data collection and team building.

To the knowledge of the authors, this is the first paper that demonstrates the use of GMB or SGMB in the animal health sector.

### 2.3 Description of study regions

Lundazi district is located in the Eastern province of Zambia and shares a border with Malawi. It is made up of a valley, which is a game management area (South Luangwa National Park) and a plateau where most people reside. Lundazi has an estimated population of 315,000 people and 25,680 households, and a human population density of 22.4 people/km^2^ [32–34]. ECF is endemic and economically the most significant disease in Lundazi and the entire Eastern province especially in the plateau, followed by African Animal Trypanosomiasis (AAT) which is mostly found in the valley [8,32].

Monze district has an estimated population of 196,000 people, and a population density of about 25 persons per km^2^ [34]. The district has three physiographic regions which include: (i) the south-eastern part of the district, that has steep slopes which borders Lake Kariba whose altitude is between 600 and 650 meters above sea level; (ii) the central high plateau area consisting of soft undulating plains, which is ideal for maize growing; (iii) the north-western low flat plain where the Kafue Flats and Kafue National Park fall which is being used for grazing cattle [35]. Cattle and crop farming are the major economic activities in Monze, and ECF is a major hindrance to traditional cattle production [35,36].

The next section comprehensively describes the process of implementing an SGMB session in these contexts.

### 2.4 Procedure of spatial group model building (case study of Lundazi and Monze districts)

#### 2.4.1 Planning of meetings

The modelling team planned the organisation of the meetings over a three-week period, which is important in order to mobilise stakeholders and successfully carry out GMB-type processes as highlighted by past research [27]. During this period, we put logistics in place, developed the agenda based on the skeleton developed by other researchers [22,37] (Table 1), invited stakeholders to a workshop, and organised a set of SGMB scripts. We gave invitation letters to the organisations that chose key stakeholders to represent them. Since Lundazi and Monze districts are respectively about 800km and 300km from Lusaka (where the modelling team is based), the gatekeepers (contact persons with the stakeholders) played a key role in inviting the stakeholders by delivering invitation letters and the agenda to them by public buses that run daily. Gatekeepers represent the link between the modelling team and stakeholders, and thus play a brokering role and control access to the community [24]. In Monze district, the gatekeeper was a former student of the University of Zambia who originally comes from there and was working at the District Veterinary Office. In Lundazi district, the gatekeeper was a Veterinary Assistant who worked at the District Veterinary Office for more than five years. We initially used both gatekeepers as enumerators during a baseline survey. Constant engagement through phone call meetings with gatekeepers and their assistants helped to make the preparation a success. The modelling team also had two brief meetings to rehearse the SGMB process. The modelling team was made up of four members; a senior SD modeller, a junior SD modeller, a rapporteur, and a gatekeeper. Other researchers have recommended larger modelling teams in GMB [37], but the lack of available expertise constrained us with the GMB and SD modelling process in Zambia. However, our smaller modelling team played similar roles as those recommended by previous GMB research [27,37].

**Table 1:** Agenda for SGMB workshop.

Time	Public Agenda	Team agenda (Themes)
08:00–08:30		Scene preparation
08:45–09:00	Registration	Participants settle
09:00–09:30	Introduction	What do we seek to achieve How we do it Concepts of systems thinking—look at whole chain/network Organisation of the session Hopes and fears
09:30–10:00	A new vocabulary-SD	Stocks, flows (intervention), parameter (variables)
10:00–10:15	**Coffee Break**	**Rearrange sitting arrangement**
10:30–11:30	SGMB process (Use of the map)	Where we are What’s there (layer definition), flip chat Who is there How it changed (setting/context) How is it changing
**11:00–11:15**	**Health Break**	
11:15–11:45	SGMB process (Use of the map)	What are key animal health issues in VC Where do they take place (local or extend?) To whom? Which most important? How to measure (variables as metrics) Behaviour over time for identified variables How to interact within context (Local process, diffusion process, structural)
**11:45–12:00**		**Rearrange sitting arrangement**
12:00–12:30	Causes of the problem	Identification of the direct and indirect (what causes the causes) Internal versus external context How does space mediate it all How interact with context?
12:30–13:00	Feedbacks	Causes and consequences How landscape mediates
	**Lunch-Day meeting End**	Debriefing
**Day 2 meeting**		
09:00–09:30	**Recaps**	What did we do yesterday? Model it
09:30–09:4	**Coffee Break**	
10:00–12:30	**Modelling and simulation**	Create simple model and simulate

#### 2.4.2 Room preparation

We prepared the scene the same day of the GMB meeting. The gatekeeper greeted and registered the participants as they arrived. The assistant facilitator (junior SD modeller) acted as an usher to organise the participant seating arrangements, which was in a C-shaped form as recommended [20,24,27]. We provided papers, pens, and markers to participants, and used flip charts, room walls, computer, and projector. We also provided tea, coffee, water and soft drinks in the room. Room preparation and registration took 35 minutes in Lundazi and 20 minutes in Monze against the planned 15 minutes because some stakeholders came late. A total of 12 and ten stakeholders (Table 2) from different organisations involved in the beef value chain attended the SGMB workshop in Lundazi and Monze districts, respectively.

**Table 2:** Beef value chain actors from which stakeholders represented.

Beef value chain actors	Monze district	Lundazi district
	No. of stakeholders	No. of stakeholders
Agricultural and veterinary input suppliers	1	2
Traditional cattle farmers	1	4
Cattle traders (intermediaries)	1	2
Beef processors	3	0
Beef retailers	1	1
Veterinary Officers	2	1
Farmers Union Non Governmental Organisation	1	1
Beef consumers	0	1
Total	10	12

Note: The consumer did not make it to the meeting in Monze district while Lundazi district did not have beef processors. The actual name of organisations where the stakeholders came from has not been included for ethical reasons.

#### 2.4.3 Script 1: Introductions, hopes and fears

The session began with personal introductions of the stakeholders. Introductions enabled the facilitator to engage the stakeholder with their surname, i.e. Mr Nkhata. The use of the first name during sessions is recommended [20], but is considered disrespectful in the Zambian culture. After introductions, the facilitator introduced the purpose of the meeting and methodology. We used the “hope and fears” GMB script [37] to assess the expectations of stakeholders from the SGMB meeting. We gave two different pieces of coloured paper to stakeholders and asked them to write one “hope” and one “fear” on each piece of paper. The gatekeeper assisted those who could not write. We taped the cards on the wall and read out. We took pictures of the cards as well as all visual exercises throughout the SGMB process.

#### 2.4.4 Script 2: An introduction to the language of system dynamics (concept model)

The senior modeller introduced the language of system dynamics using the concepts of stocks, flows, and parameters. The language of system dynamics was introduced early in the session through the use of simple concept models (like the one in Fig 1), since stakeholders were unfamiliar. The concept models helped stakeholders to visualise the main objective of the activity and to use SD terminology effectively in model development [37,38]. The concept models were drawn on the flip charts and explained to the stakeholders in a local language. The stakeholders understood the concepts quickly and were able to define the concepts of stocks, flows, and variables in the local languages (Tumbuka and Tonga). This session was followed by a tea break, during which the facilitation team re-organised the seating arrangements for the next exercise on SGMB.

#### 2.4.5 Script 3: Spatial group model building using LayerStack

In this session, the stakeholders stood around the table where the facilitating tool, LayerStack (Fig 2), was placed. After introducing the session, we placed the map of the study region in a transparent sleeve to start the group exercise. Six transparencies (layers) represented different categories of information and were placed on top of the map: (i) settlements, (ii) value chain actors and supporters, (iii) cattle production, (iv) cattle sales, (v) disease and (vi) household socio-economic status. Stickers of different colours and shapes were used to represent actors and processes along the beef value chain in Lundazi and Monze districts (Fig 2). We plotted behaviour over time (BOT) graphs to illustrate the reference modes (baseline dynamic behaviour) [37] of the dynamic context of different system phenomena on the layers of the facilitation tool. Behaviour over time graphs focus on patterns of change over time of variables as opposed to isolated events and help to hypothesise causal relations [39]. At first, the stakeholders felt uncomfortable with the exercise, but within 10 minutes, they became active and enjoyed the exercise, which elucidated many interesting socio-economic, cultural, and ecological contextual issues. The session lasted 120 minutes on average and included one break. There were variations in two study regions with regard to the time taken to grasp SD concepts. Traditional cattle farmers in Lundazi grasped the systems thinking language quicker than those in Monze, which we may attribute to the facilitator’s fluency in local languages. The facilitators used English more in Monze and a local language (Tumbuka) more in Lundazi district.

#### 2.4.6 Script 4: Problem identification (feedbacks)

The first day’s last session focused on problem identification, its causes, and consequences. The facilitator introduced the session with a description of feedback loops in system dynamics. Since we had already identified the problem of ECF outbreaks in the baseline survey [9], this part of the exercise had stakeholders validate the problem. Stakeholders validated the problem by carrying out a GMB exercise on “causes and consequences” associated with the problem [20]. We gave blank pieces of paper to stakeholders to write the causes and consequences of ECF. The causes and consequences exercise involved asking the stakeholders to identify the cause of the problem, its consequences and the effects of the consequences, until coming back to the original cause. We taped the papers on the wall after consensus was reached. Where different opinions existed, voting through the use of hands was done, and a majority view was adopted (consensus). The facilitators, gatekeepers, and recorders actively engaged the stakeholders to participate. We drew feedback loops on the wall. The session took 60 minutes and was followed by a concluding lunch. The stakeholders dispersed after lunch, but the modelling team remained to de-brief as recommended [28].

#### 2.4.7 Script 5: Modelling and simulation (day two meeting)

On day two, the stakeholders were engaged in model development based on the SGMB sessions on day one. We asked the stakeholders to identify the stocks, flows, and variables based on what we discussed during the previous SGMB sessions. Through this process, the stakeholders identified and developed four key models that influence the dynamics of ECF: (i) household socio-economic status model, (ii) SIR epidemiological model, (iii) vector-parasite model, and (iv) land use model (to be discussed later in the text). Draft models were developed in real time with stakeholders using Stella Professional software and beamed on the wall through the projector. To exemplify the usefulness of the approach, the household socio-economic module was directly parameterised by the participating stakeholders using hypothetical values since stakeholders did not have all parameters available or adequate scientific information of some parameters in the models. The senior modeller ran simple simulations to provide participants with an appreciation of how the SD models can be used to understand how different policies could influence ECF over time. This process generated interest from the stakeholders because their qualitative discussions were converted into models that could help with decision-making. The modelling session closed by midday. The modelling team informed stakeholders that they would be presented with models for validation once the formal models were fully developed and parameterised (through additional key informant interviews and literature review). Parameterization requires several rounds of meetings, a literature review, and interviews with key informants, which requires resources that were not available for this initial pilot exercise.

## Results and discussions

**Fig 3:**
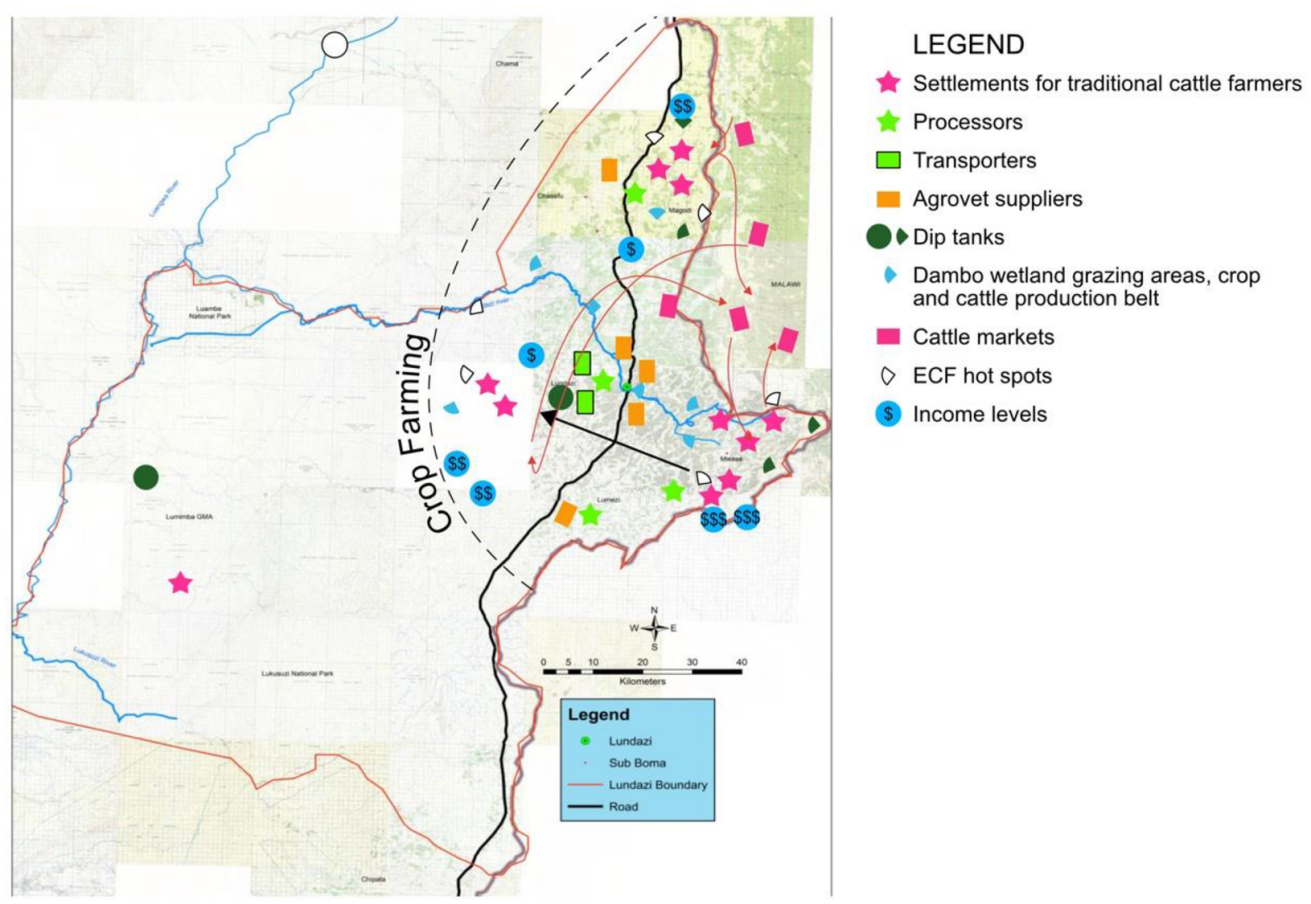
Map of Lundazi District based on the LayerStack process (developed by authors). Reprinted from [Zambia_Mosaic_250Karc1950_ddecw] under a CC BY licence, with permission from the Surveyor General, Government Republic of Zambia, original copyright, [1974].

**Fig 4:**
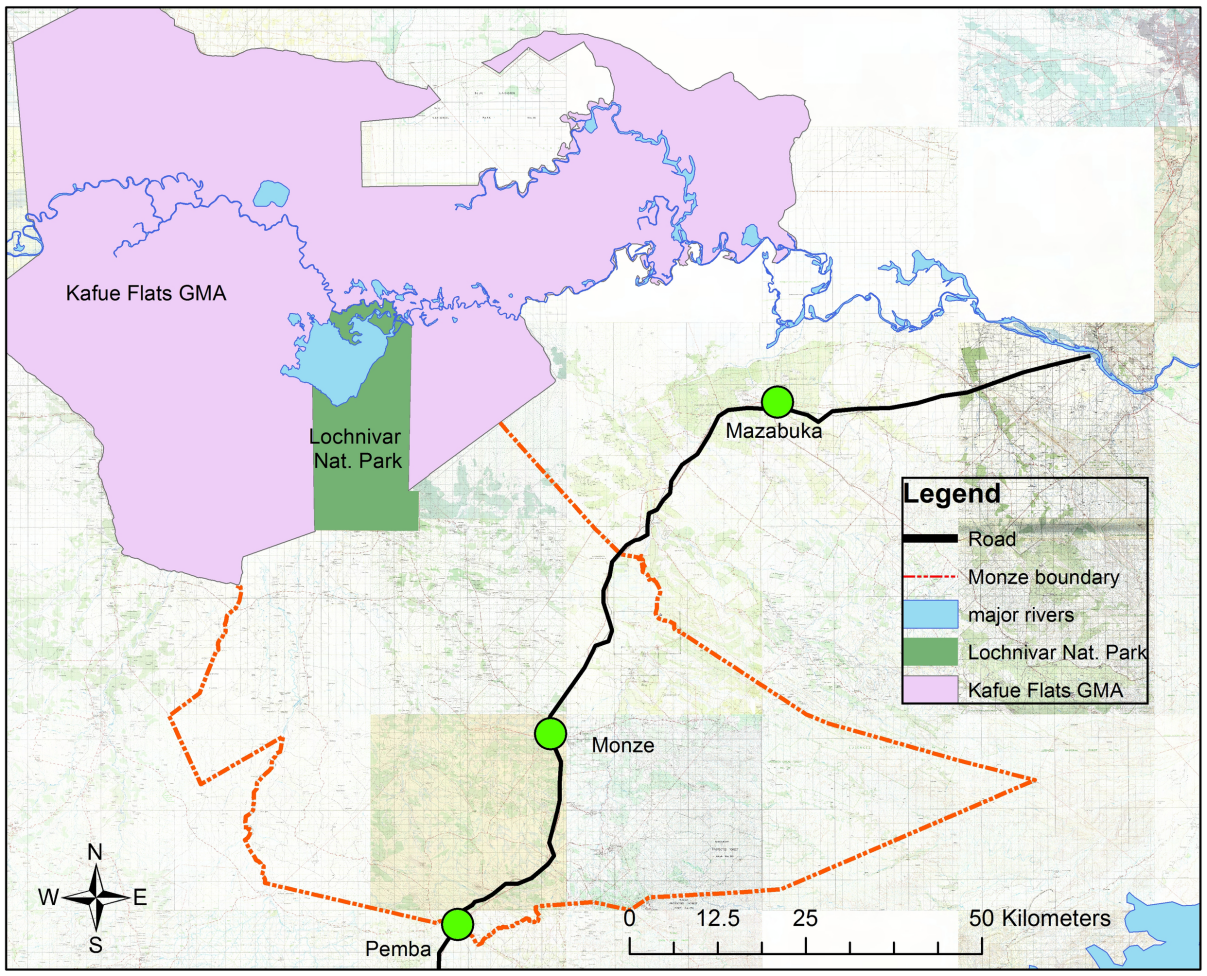
Map of Monze District. Reprinted from [Zambia_Mosaic_250Karc1950_ddecw] under a CC BY licence, with permission from the Surveyor General, Government Republic of Zambia, original copyright, [1974].

The following sections describe (1) a discussion of the SGMB input (modules), (2) process of model building and model structure, and (3) a discussion of learning outcomes from the SGMB process, feedback loops, and SD mental models. The utility of LayerStack is highlighted in Fig 3, which illustrates a digitisation of the layers and results from the SGMB discussion in Lundazi district and will be referred to throughout this section. We did not have sufficient graphical resolution with the map from Monze to digitise layers similarly. However, we do provide a base map, (Fig 4). We describe and contrast some of the differences between Monze and Lundazi districts in the discussion below.

### 3.1 Discussion of SGMB modules

#### 3.1.1 Spatial overview of the study regions

In Lundazi, SGMB discussions demonstrated that cattle production takes place in the plateau (eastern part) with very little activity in the valley (western part) due to the presence of tsetse flies that cause Trypanosomiasis (Fig 3). The eastern part of Lundazi in the plateau is the most densely populated human settlement (marked with a pink star in Fig 3) and hence has the highest number of traditional cattle farmers followed by the north-eastern part. However, cattle farmers in eastern part have been migrating to other areas such as the western (shown by a black arrow in Fig 3) and north-eastern parts of Lundazi, due to depleting land resources. Migration out of the settlements has therefore been increasing over time. The migration to new areas could be the reason for new ECF hotspots to areas which were initially disease-free. This finding justifies the need for a spatial approach to ECF interventions.

The SGMB process in Monze revealed that cattle grazing mostly take place in the North-western part of Monze near the Kafue flats (floodplain) and Lochinvar National Park (Fig 4). The stakeholders reported that a transhumance cattle herding system is practised, with the movement of cattle between the Kafue flats and the plateau around the central part of Monze. Human settlements are found more in the plateau where maize is being grown, and cattle only come in or near when the Kafue flats get flooded during the rainy season.

#### 3.1.2 Value chain actors

The SGMB session in Lundazi revealed that there is only one slaughter facility in Lundazi central business district (CBD). Three slaughter slabs exist in major cattle producing areas in the eastern part of Lundazi with all of them being owned by the local authority. Stakeholders reported that the local authority is not allocating land for the construction of abattoirs which makes the construction of more slaughter facilities a challenge. The number of processors (marked with a green star in Fig 3) has, therefore, remained constant despite population growth in the district. However, the number of retailers (butchers) is on the increase. Demand for beef is rather seasonal and strongly dependent on income received by civil servants (who are paid at the end of the month) and crop farmers who mostly purchase beef in the dry season after harvest when they sell crops. Stakeholders reported that April and May are months of highest sales of beef (after farmers sell their crops). There is no large-scale processor in Lundazi district, and thus cattle farmers sell their beef cattle directly to butcheries or slaughter locally and sell to fellow farmers and villagers on credit to pay after the harvest of crops.

In Monze district, the SGMB process revealed that all beef value chain actors apart from cattle producers are found in the central business town (CBD). There are four big beef producers in Monze district with a more organised retail and consumer base as compared to Lundazi district. This is because Monze district has farmers who own large herds of cattle. The beef processed by these large-scale processors is mostly meant for the market in Lusaka and the Copperbelt provinces where it fetches a better price, with small quantities being supplied to the local retailers through their organised butcheries (vertical integration). Small-scale retailers (butcheries) mostly access their beef through informal markets.

Lundazi district has seven agro-vet suppliers (marked by the orange box in Fig 3) that exist in the CBD and four in the eastern rural part where cattle production takes place. Common drugs purchased include Oxytetracycline (commonly known as Oxyject), Procaine Penicillin (commonly known as Megapen), Ivermectin (commonly known as Ivomec) and Albendazole deworming tablets. East Coast Fever, locally known as “*kandukutu*” is the main chief complaint presented when purchasing drugs for cattle. Consumption of ECF drugs has been increasing over time. However, the increase in Lundazi is seasonal and is driven by income availability (higher in the dry season after farmers sell their agro products). All participants agreed that the availability of drugs was not a huge challenge but the availability of income hindered affordability.

Monze district has more agro-vet suppliers who supply a wide range of products than Lundazi. Stakeholders reported that the large cattle population is a driver for investment into agro-vet products as most of the investors target Southern province due to the presence of the largest number of cattle in Zambia. However, all of these agro-vet shops are in the CBD which makes availability in rural areas a challenge. This gives room for intermediaries to buy the drugs and trade them at a higher price in rural areas, thus hindering affordability.

There are no non-governmental organisations (NGOs) that are directly promoting the traditional cattle subsector in either Lundazi or Monze districts. The government through the Ministry of Livestock and Fisheries are the only ones working directly to support the beef industry in Lundazi although the support is more restricted to policy formulation.

In Lundazi district, only a few transporters (green box in Fig 3) exist because of low demand for cattle transportation services as traditional cattle farmers walk their animals to slaughter facilities in Lundazi and across the border into Malawi. By contrast, Monze district has a huge number of transporters due to the thriving beef and dairy cattle subsectors, coupled with the distant distribution of farmers.

#### 3.1.3 Production

The SGMB session revealed that traditional cattle farmers in Lundazi district practice a village resident cattle herding system because the availability of grazing areas is a challenge. It was reported that farmers graze their animals in their fields after harvesting crops. A few communal grazing areas in the form of dambos (wetlands), marked by a blue pie shape in Fig 3 exist along the Lundazi River, Lake Bell, and Lumezi River. These are favoured grazing areas for farmers mostly in dry season. Availability of grazing areas in Lundazi is a challenge that has forced farmers to keep smaller herds of cattle. In Monze district, by contrast, stakeholders reported that cattle grazing is not a problem due to the presence of the Kafue flats (north-western direction on the map in Fig 4) which has abundant grass even in the dry season.

Stakeholders reported that there are 21 dip tanks (marked as the green circle/pie shape in Fig 3) in Lundazi district but only one is functional. Dipping depends on the availability of income for farmers to buy acaricides and service dip tanks. Agro-vet shops stock dipping chemicals and encourage farmers to dip their livestock. Farmers reported that they are willing to dip their animals. However, the distance to the dip tanks present a challenge because they have to seek permission from other farmers to pass through arable land where crop farming activities such as maize and soya beans are carried out. Animal-crop conflicts are thus increasing due to human population increases which have led to greater demand for crop farming. Thus, as the human population increases, crop production increases while dipping falls because passage through arable land is a challenge. Few farmers in Lundazi have resorted to using sprayers to control ticks and other parasites. Tick activity is reported everywhere in the district. High tick activity is experienced during the rainy season (i.e., in March) when crop production is at its peak, and dip tanks are non-functional and inaccessible. Animal-crop conflicts are therefore a driver of ECF and other tick-borne diseases.

In Monze district, it was reported that there are dip tanks in the plateau (central part of Monze in Fig 4) and very accessible, but the management of these dip tanks has been a problem as there are no viable committees or farmer groups to manage them. The stigma associated with a social class where those with more cattle (rich) do not want to mix with those with fewer cattle (poor) further complicates ECF control efforts. However, there is no access to dip tanks in the Kafue flats where new ECF hotspots are developing. This makes it difficult for farmers to dip their animals during the period when animals are in the Kafue flats. Stigma due to social class is not a problem in Lundazi due to even distribution of cattle.

#### 3.1.4 Cattle sales

In Lundazi district, farmers sell their cattle to butchers and other farmers. A government-owned market exists in the Lundazi CBD, but it has been non-operational for the past ten years. Butchers characteristically follow the farmers to make their purchases, making cattle sales more farm-gate (cattle sales from the farm) in nature. The behaviour over time of farm-gate sales has been relatively constant. There are viable cattle trading activities in Malawi (a neighbouring country) in areas such as Jenda, Phwazi, Lilongwe, Bulala and Embwangweni (pink boxes in Fig 3). This trade is informal with cattle movements not regulated by the veterinary department and customs office. The veterinary department neither encourages nor condones such acquisitions; while illegal, the absence of formal markets continues to drive this form of cattle trade. The exchange rate of the Malawian Kwacha to the Zambian Kwacha also influences this trade. There are no border provisions for cattle export/import fees. The behaviour over time of sales oscillates with peaks between November and February and troughs between April and July. The potential market for beef in the neighbouring Chipata district has not been exploited because of the formal process involved in the movement and transportation of cattle which increases transactions costs.

In Monze district, cattle marketing was reported to be mostly local due to the presence of four large processors and many intermediaries who buy cattle in the Kafue flats and surrounding villages. The informal movement of cattle outside of Monze in search of lucrative markets in Lusaka and Copperbelt provinces were reported by stakeholders, although to a lower extent as compared to Lundazi district. Drivers of cattle sales in both Lundazi and Monze districts include the Government Farmer Input Support Program (FISP) season, school fees, and household needs. Therefore, cattle sales were seasonal with peaks in January, April, August and December. This is in line with the national school calendar and crop farming season. An average of four cattle per year per farmer are sold in Monze as compared to only two in Lundazi district. As noted earlier, these sales are seasonal and driven by pressing household financial needs, school fees, and farm inputs for crop farming.

#### 3.1.5 Diseases

Stakeholders in Lundazi district reported that calves are of high importance in the local context, given by the local slogan “*Ng’ombe ni ma thole*” meaning “Without calves, there is no herd of cattle”. Stakeholders reported that ECF is responsible for most calf mortalities. The ECF immunisation program offered by the government has been very beneficial and has contributed to the reduction in mortalities among farmers who can afford to pay for ECF immunisation services. Other diseases include Trypanosomiasis (Kaungu), Lumpy Skin Disease (LSD). East Coast Fever hotspots (marked by the white/translucent pie shape in Fig 3) are found in areas in the plateau. The behaviour over time of ECF cases is decreasing among high-income farmers (marked by $ $ $ in Fig 3) who can afford ECF immunisation, and increasing among those with low and medium household socio-economic status (marked by $ and $ $ in Fig 3).

In Monze district, stakeholders reported that ECF cases are normally reported in the plateau (central part of Monze in Fig 4) where people cultivate their crops. None or few cases were reported in the Kafue flats near the game management areas (livestock-wildlife interface areas) because *Theileria parva* does not complete the life cycle due to floods [2,36]. However, stakeholders reported that cases of ECF are now increasing exponentially due to fewer floods in grazing areas which are caused by less rainfall as a result of climate change. This dynamic could reduce the effectiveness of current ECF control efforts in the future.

#### 3.1.6 Socioeconomic status

The SGMB sessions in both settings revealed that areas with high cattle density also have more people with a higher socioeconomic status (marked by blue dollar signs in Fig 3). The eastern part of Lundazi district is near Malawi (< 10Km) and hence engaged more in trade-related activities. Farmers in these areas were reported to have a higher socio-economic status because they have more cattle othen others, and hence can cultivate larger portions of land (50–100 ha) than those without cattle (who only cultivate up to 2ha). This is the same scenario in Monze district. The gap between the rich and the poor is increasing. Likewise, the cattle population for farmers with higher socio-economic status is also reported to be increasing. Farmers with more cattle can pay for ECF drugs. Attitudes towards the purchase of drugs and income availability are important drivers of expenditure on ECF drugs. Stakeholders noted that everyone is vulnerable to livestock diseases, but the vulnerability is less among individuals with higher incomes and socio-economic status.

### 3.2 Model building process and model structure

**Fig 5:**
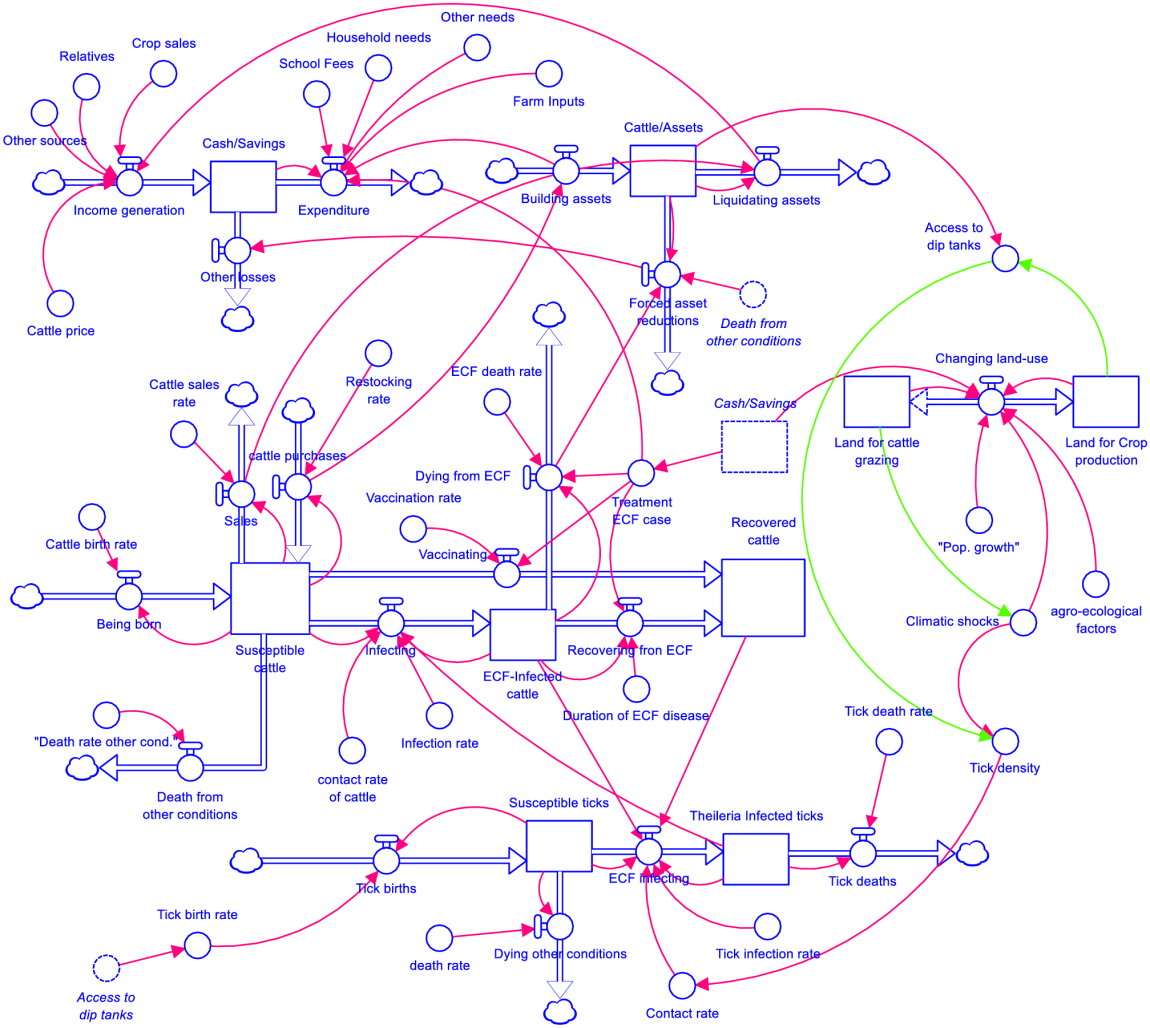
System dynamics mental models (developed using Stella Professional).

After the SGMB process, we built a conceptual SD model that involved the identification of the stocks, flows, and variables that describe important system phenomena. Three key drivers of ECF were identified by the stakeholders and the modelling team in Lundazi and Monze districts: household socio-economic status, land use, and disease dynamics (spread across cattle populations and host-parasite interactions). These drivers were incorporated into different, interacting SD modules that are described below and summarised graphically in the stock-flow diagram in Fig 5.

#### 3.2.1 ECF epidemiological model

The ECF epidemiological model is a representation of a simple SIR (susceptible-infected-removed) model commonly used in the epidemiology literature [40]. In a SIR model, animals pass through three possible states over the course of a disease outbreak. They begin as susceptible to a disease, with one animal (or more) initially infected with the disease. Animals move to the infected state based on contacts with other infected animals and the degree of infectivity of a disease pathogen. Animals move from infected to recovered state based on the time taken to recover from the disease.

In Fig 5, we represent these three states through stocks denoted susceptible, infected, and recovered cattle. These stocks represent the number of animals found in each state at any given period of time. In the case of ECF, vaccinated or immunised cattle (though infection and treatment method, or ITM) can be considered as analogous to being naturally infected and recovered. This explains why we did not add a separate vaccination stock, although we did consider a flow from susceptible to recovered to consider vaccination via ITM. However, ECF vaccination also depends on income availability just like treatment and tick control. The thick arrows between states (flows) represent the infection of susceptible animals (the flow “infecting”) and recovery of infected animals (“recovering from ECF”). The model also includes inflows of animals born or purchased over time, and outflows of animals sold or which die (either naturally or from ECF), all of which are mediated by technical parameters that define birth rates, purchase rates, sales rates, and death rates from ECF and naturally.

In parallel with the dynamics of disease within the cattle population, we model parasite (tick) infection dynamics and host-parasite interactions. The tick model has two stocks: susceptible ticks and infected ticks, and four flows; tick birth, tick-infecting, and tick death, and several parameters influencing each flow (Fig 5). The parasite is *Theileria parva* and, the vector is *Rhipicephalus appendiculatus*. Climate, host availability, and vegetation determine vector survival [16,17,36]. The epidemiology of ECF is dynamic with a complex transmission which relies on a number of factors such as strain pathogenicity, pathogen load, and the immunity of the host for the tick to be infected. As such, not all infectious ticks transmit disease when feeding on uninfected hosts, and not all uninfected ticks feeding on infectious host become infected [17]. Four scenarios exist in the tick population, namely: detached non-infectious tick, detached infectious ticks, attached non-infectious, and attached infectious ticks [17] which we have also depicted in our parasite model. Therefore, tick infection is a product of the susceptible ticks, infected ticks, infected cattle, recovered cattle, contact rate and infection rate. Contact rates depend on the tick density which was influenced by land use and climate change (Fig 5). Spatially, some areas are more prone to tick breeding due to enabling environment factors, and this increases the stock of susceptible and ECF piroplasm infected ticks.

Likewise, for an infection to occur in cattle, there must be the presence of an infected tick, infectivity of the ECF piroplasm, and contacts between the susceptible and the infected stock (cattle). In the model, animals can be treated to promote recovery from ECF, but this will depend on the ability of farmers to treat based on their income (see next section). Three scenarios exist in the ECF SIR model: susceptible asymptomatic cattle due to low threshold for transmission to ticks, symptomatic infectious cattle that transmit the pathogen to ticks, and recovered asymptomatic but with a proportion of which are still infectious and can transmit the pathogen to ticks [17,41].

#### 3.2.2 Household socio-economic model

From the SGMB sessions, we overlaid the epidemiology of ECF alongside socio-economic considerations. We considered socio-economic issues through constructing a simple model of household cash flow and asset accumulation. Households are assumed to have a stock of cash whose level at any period of time depends on inflows of income and outflows of expenditures. The sources of income (determinants of income generation rate) for traditional cattle farmers were crop sales, cattle sales, relatives and other activities. Cash is mainly spent on school fees for children, household necessities, farm input for crop production, and other needs. While not explicitly highlighted in the conceptual SD model, both income and expenditures are both spatially and seasonally determined as noted in the discussion in section 3.1. In particular, certain sources of income and expenditure will vary depending on the time of year, while the timing and volume of income generated from crops or cattle will vary by region. Thus, while the structure of household factors will be similar in Lundazi and Monze, their relative effects quantitatively could be quite different.

Households also keep a stock of physical assets (mainly cattle, but also other infrastructure). Assets can be acquired through the purchase of cattle and liquidated through their sale. Consequently, this will have an impact on their cash flow—buying cattle will result in greater expenditures, while selling cattle will provide a boost in income. Assets can also be forcibly liquidated due to disease or the death of animals, providing implicit cash loss (in opportunity cost terms) for farmers.

The household model is linked to the SIR model through sales, purchases, and deaths of animals. These factors in the SIR model influence the asset composition of farmers, which in turn influence the cash availability of farmers. The amount of cash available for farmers has a feedback effect in terms of the ability of farmers to spend money to treat, dip (access to dip tanks) or vaccinate animals against ECF. Where cash availability is low, this limits the ability of farmers to treat ECF-affected animals, causing more stock losses, a reduction in farm assets, and further losses in income which prevent farmers from treating animals in future. This reinforcing feedback loop highlights the vicious cycle that farms often face in trying to treat animals for ECF during ECF outbreak events, which can be compounded further by seasonal shocks to household income (e.g., the need to pay school fees or other livelihoods needs), which are regionally and seasonally mediated [42,43]. On the other hand, more consistent sources of regular income or buffers from formal credit or insurance markets could help to mitigate these shocks, but these types of risk mitigating devices are often limited or non-existent in rural Zambia.

#### 3.2.3 Land use model

We also considered the role that land use patterns play in our system, both in terms of the role that different land types play regarding disease dynamics and income generation. The simple land-use model has two stocks (land for crop production and land for grazing), three flows (land use) and several parameters (Fig 5). The model demonstrates how landscape influences the pattern of ECF disease dynamics. Under the causes and consequences session, the stakeholders reported climate change and land-use due to population growth to play an important role in ECF disease occurrence.

The land use model is connected with the parasite model through two feedback loops, one for Lundazi district and the other one for Monze districts (represented by green arrows in Fig 5). In Lundazi district, there is a balancing feedback loop, reduced access to dip tanks increases tick density which eventually increases the contact rate between the ECF-infected and non-ECF-infected ticks. In Monze district, by contrast, there is a reinforcing ecological feedback loop. Increased rate of drought in the Kafue flats increases tick density which also increases the rate of contact between ECF-infected and non-ECF-infected ticks. From a policy standpoint, there is value in reducing the tick density between the infected and non-infected ticks through increasing access to dip tanks and creating awareness against the stigma in the use of communal dip tanks.

Land use among traditional cattle farmers in both districts is dual purpose for cattle grazing and crop production. In both districts, crop production is the major income generating activity. When prices for crops increase, traditional cattle farmers produce more volumes of crops making less availability of land for cattle grazing. Therefore, price effects determine profitability which is an important driver of changing land use. Population growth also influences change in land use. The higher the population, the higher the demand for food, and the greater the need for land for crop production.

Agro-ecological zones and disease dynamics also influence the dynamics of land use. Traditional cattle farmers tend to avoid areas that are prone to disease. In Lundazi district, cattle farmers reported that they avoid keeping cattle in the valley (western part in Fig 3) because of the high prevalence of African Animal Trypanosomiasis. Such agro-ecological zones, therefore, would only be suitable for crop farming. Similarly, the south-eastern part of Monze district, which is made of steep slopes bordering Lake Kariba and whose altitude is between 600 and 650 meters above sea level, does not support viable livestock farming.

In Monze districts, climatic shocks influence changes in land use, e.g. when there is drought, farmers graze their cattle down into the Kafue floodplain which was initially not accessible due to floods. Climate change, therefore, provides a suitable environment for the survival of ECF tick vectors and creates new hotspots.

### 3.3 Discussion of the learning outcomes from the SGMB process, feedback loops, and SD models

The SGMB process and initial conceptual SD model have brought out a number of interesting aspects which many researchers, local decision-makers, and other stakeholders in animal health tend to overlook when designing and implementing disease control policies. For example, in this process, we learned that there is considerable diversity in the dynamic context of ECF due to differences in spatial patterns, and thus, policies may not be equally effective across different areas. ECF interventions (through tick control) in Lundazi are hindered by competition for land use between crops (especially maize, a staple food) and cattle (used for draught power to cultivate more crops). This has made the use of dip tanks obsolete, yet they are critical in establishing enzootic stability (keeping the host, vector and pathogen stable). In Monze district, by contrast, the model structure is influenced by the effects of climate change. Climate change has led to droughts and fewer floods that would otherwise provide an enabling environment for ticks and the ECF parasite to thrive. Strategic immunisation and placement of dip tanks (control of ticks) and establishing of veterinary infrastructure in cattle producing areas would help to change ECF system behaviour.

The contrasts in access to cattle markets between the districts also influence ECF disease dynamics. For example, in Monze district the market is more internal while in Lundazi district the market is more externally-focused. The external influence of the cattle market in Malawi does complicate effective ECF control interventions through the introduction of naive cattle that are susceptible to ECF. Cattle from Malawi may also introduce a new strain of the parasite which may further change ECF system behaviour. Internalising cattle sales through a creation of stable and sustainable markets in Lundazi district is a potential policy leverage point towards successful ECF interventions.

Variations in cultural shocks also influence ECF system behaviour. The stigma associated with mixing of animals for traditional cattle farmers of different social classes in Monze further complicates disease control efforts. These are the unintended consequences of the social class created through herd sizes where the gap between the poor and rich keeps widening, as reported in Monze district. The effects of stigma on dipping imply that ECF is not only biologically determined but also socially constructed and maintained just like in the case of anthrax in the Western Province of Zambia [44]. Remedying these social conflicts is a policy leverage point for a more effective intervention of ECF in Monze district and other areas with similar trends.

Variations in agro-ecological zones and cattle herding practices also influence ECF system behaviour. Monze district practices transhumance cattle herding system in human-wildlife-livestock interface areas. The contact between cattle and wildlife in this system further complicates ECF disease control efforts [45,46]. These variations are also important for formulating locally relevant policies.

## Conclusions and policy recommendations

The spatial aspects and interactions of socio-economical, cultural, and ecological drivers play a critical role in designing and implementing effective and sustainable community-led ECF control policies. The SGMB process and SD models developed by stakeholders demonstrated the dynamism and complexity of ECF control. We argue that ECF control is unattainable with the current control efforts which do not consider the social and landscape context of this complex and dynamic animal health problem. These issues vary considerably across space and context, suggesting that a one-size-fits-all policy will not be effective. Therefore, policy design and implementation must consider local needs for effective disease control interventions.

The demonstration of system dynamics modelling and SGMB’s applicability in addressing complex and dynamic animal health problems is a good first step towards more effective disease interventions. We, therefore, recommend the use of participatory epidemiology and systems thinking approaches to address animal health problems for stakeholders to create buy-in for bottom-up led disease interventions. Involving stakeholders in policy design could improve the uptake of interventions [23].

Finally, further research is needed to more fully parameterise the qualitative SD mental models developed in this paper to quantify the dynamic behaviour and unanticipated consequences that the highlighted socio-economic and ecological drivers have on the complex and dynamic ECF systems. Nonetheless, the qualitative analysis and use of SGMB principles show the value that participatory techniques can play in improving our awareness of the drivers of disease and non-technical considerations that may influence them.

### Ethical considerations

We obtained ethical clearance (consistent with Norwegian University of Life Sciences policy) from Excellence in Research Ethics and Science (ERES) Converge, reference number “2016-Nov-006”
